# A randomized controlled trial of the effects of dog-assisted versus robot dog-assisted therapy for children with autism or Down syndrome

**DOI:** 10.1371/journal.pone.0319939

**Published:** 2025-03-19

**Authors:** Steffie Van Der Steen, Erica Kamphorst, Richard E. Griffioen

**Affiliations:** 1 Department of Pedagogical and Educational Sciences, Faculty of Behavioural and Social Sciences, University of Groningen, the Netherlands; 2 Department of Entrepreneurship, Business, Animals & Food, Aeres University of Applied Sciences, Dronten, the Netherlands; Federal University of Paraiba, BRAZIL

## Abstract

Research with controlled or crossover designs in animal-assisted therapy have largely used control groups receiving no treatment or treatment as usual, which can potentially inflate the effects of these interventions. It is therefore not always clear whether this type of therapy has a positive effect on, for example, the social skills of children with special support needs. To address this, the current study compared children (7-16 years, *n* =  65) with autism or Down syndrome who received five sessions of dog-assisted therapy (*n* =  24; 9 female) with an active control group who received five similar sessions of robot dog-assisted therapy (*n* =  21; 8 female) and a no-treatment control group (*n* =  20; 8 female). The robot dog was capable of performing autonomous behavior and responding to the child’s actions and verbalizations. Using parental questionnaires, we assessed children’s social and emotional skills before and after the therapy sessions or waiting period and included a follow-up measurement after 4-6 weeks. On a group level, multilevel analyses showed that children who received dog-assisted therapy improved significantly more in terms of emotional attunement and emotion regulation than children in the two other conditions. No significant differences were found for social confidence, conversational attunement, social cognition, and social motivation. Change from post-test to follow-up was also less apparent. Yet, on a more individual level, when looking at the Reliable Change Index (RCI), most of the highest RCIs (within the 90^th^ percentile) were found in the dog-assisted therapy group. In contrast, most of the lowest RCIs (within the 10^th^ percentile) were found in the robot-assisted group. We discuss the pros and cons of a more individualized approach in this field of study and propose a possible alternative by focusing on interaction dynamics.

## Introduction

Animal-assisted interventions aimed at improving clients’ behavioral or social-emotional functioning have grown rapidly in popularity over the past decade. Animals are now included in various educational, coaching, and therapy settings worldwide [1]. In animal-assisted therapy specifically, animals play a role in the therapeutic process as part of a structured, targeted intervention by a trained clinical or health professional [2]. Although the role of animals in therapeutic settings can be diverse [3], they are mainly included for their companionship and calming effect [4], but also to promote the social skills of the client. Some authors have argued that animals can act as “social catalysts” stimulating interactions with other people [5], and provide clients with the opportunity to improve their communication skills and adapt to interactions with the animal, which can be later transferred to human interactions [6–9].

From the very beginning, animal-assisted therapy has been a popular intervention for children with developmental difficulties or who face social challenges [1,10]. Children seemingly have a natural connection with animals and quickly bond with them, as evidenced by numerous studies on the benefits of pet ownership for children [11,12]. Especially populations that are more difficult to support through interventions that rely on verbal interactions and metacognitive skills, such as children with intellectual disabilities such as Down syndrome (DS), or children with autism spectrum disorder (ASD; hereafter referred to as autism), animal-assisted therapy quickly became a logical and well-considered choice in practice [13].

In the eyes of the public, the therapeutic value of interactions with animals seems evident [1]. It took a while for researchers, however, to initiate large-scale studies in this particular area. After initial studies that used smaller sample sizes or lacked a control group, research in this area has now blossomed [14,15]. Between 2016 and 2020 alone, more than 43 empirical studies on animal-assisted therapy specifically for children with autism were published [8], as well as several literature reviews [16–17] and meta-analyses [18–19].

Whereas earlier literature reviews and meta-analyses [20] clearly indicated the need for more randomized control trials (RCTs), several RCTs and studies with controlled or crossover designs have now been conducted. For example, one study [21] found a significant positive effect on the adaptive social and communication skills of young children (aged 3-8, *n* =  73) with autism who underwent a biweekly 4-month dog training intervention, compared with children in the waitlist-condition. A study with slightly older children with autism (aged 8-14, *n* =  31) compared the results of a 12-week social skills training with and without a dog. Children in the dog-assisted condition had higher teacher report scores on social behavior and reported fewer interpersonal problems [22]. In a systematic review of animal-assisted interventions for people with an intellectual disability, nine out of ten studies reported improvements in social interactions and communication skills [23]. A smaller study which evaluated the effect of a 6-week dog-assisted program for children with DS and autism (aged 11-18, *n* =  10), reported a decrease in emotional and behavioral problems for both groups, with a bigger, non-significant decrease for children with DS [24].

It is important to note, however, that research with controlled or crossover designs in animal-assisted therapy have largely used control groups receiving no treatment or treatment as usual. This can potentially inflate the effect of animal-assisted therapy, leading several authors to advocate the use of “active control groups” undergoing comparable interventions [5,20,25]. The idea is that being immersed in a new (ideally similar) intervention as part of an active control group, with new aspects that are different from ‘treatment as usual’ or being on the waiting list, can promote motivation and positive feelings. Any differences between an active control group and animal-assisted therapy seem to indicate more credible evidence if they are in favor of the latter [5,20].

The inclusion of robots allows researchers to compare animal-assisted therapy to such similar active control groups. While every play store has a selection of stuffed animals that make sounds or move, more advanced robot animals are highly interactive and can perform similar actions as live animals [26–28]. For instance, some robot dogs act in a similar way as living dogs by showing autonomous behavior and are equipped with sensors to enable them to react to their environment [29]. Research on the well-known robot dog AIBO (Sony) shows that children (aged between 7 and 15) treated it as a real dog, although they were more likely to attribute traits such as sociality and moral sense to a real dog [30]. Other studies show that children regarded the same robot dog as a technical pet and often attached the word ‘dog’ to it, while adults were more likely to compare it to a machine [31]. Ribi and colleagues [27] compared 3-6 year old children’s interactions with a real dog with the robot dog AIBO, and found that children initiated contact with the robot dog more often than with a live dog, although a higher percentage of children refused contact with the robot dog (30.5% compared to 18.2% of interaction refusals with the real dog).Although successful interventions with robot dogs and other robot animals, such as the Paro robot seal, have been conducted with patients with dementia [32], as well as some pilot studies with children with autism [29,33], therapists who work with animals have mixed feelings about the inclusion of pet-like robots [34]. On the one hand, therapists have noted that robot dogs could be a beneficial preparation or even a promising alternative for animal-assisted therapy in the case of allergies or fear of dogs. On the other hand, therapists have commented that robot dogs cannot replace the real human-animal bond, comfort, and social learning experiences that clients can have with real dogs [34]. This line of reasoning illustrates why an intervention with a robot dog could be the ideal active control group when investigating the effect of dog-assisted therapy. Much like real dogs, robot dogs can function as a social link between therapist and child. More importantly, with a robot dog one could perform a series of therapy sessions exactly similar to therapy sessions given with a live dog, provided that the robot dog is capable of performing autonomous behavior and able to react to what’s going on in the environment [33].

Therefore, in this article we used a RCT to compare the effect of five sessions of dog assisted therapy and robot dog-assisted therapy on the social skills of children with DS or autism. The main research question guiding this study was: To what extent does dog-assisted therapy influence the social behavior and skills of children with autism or DS, and is there a difference between (individual) therapy with a real dog, (individual) therapy with a robot dog, and a no-treatment control group? In line with the idea that robot dogs cannot replace the real human-animal bond and social learning experiences during therapy [34], we hypothesized that the effect of therapy with a real dog on the social skills of children with autism or DS would outweigh the effect of therapy with the robot dog and naturally occurring development of the no-treatment control group.It is important to keep in mind, however, that the highly regarded scientific evidence emerging from RCTs does not automatically translate to individual children that therapists in the field work with. Even when the sample is representative of the larger client population, the findings may not translate directly to a particular client or the client-therapist-dog triad [35]. Therapists interested in evidence-based interventions must decide on individual treatments using group data from studies, which show varying outcomes in the field of animal-assisted interventions [15]. To address this, we also exploratively analyzed the characteristics of children who benefited most and least from the dog-assisted and robot-dog-assisted sessions.

## Materials and methods

### Design

Children with autism or DS were matched on gender, age, and diagnosis, and then randomly assigned to a dog-assisted therapy condition or a control condition (waiting period) using a random number generating formula in Microsoft Excel. Children participating in the robot-assisted therapy condition were recruited separately at a later date for logistical reasons. A social skills questionnaire for parents was administered before the start of the intervention or waiting period, immediately afterwards, and 4-6 weeks after the intervention (follow-up). This study is part of a larger study approved by the ethics review committee of the host university (approval number: PED-1819-S-0001) and pre-registered with the Open Science Framework (https://osf.io/bc4y2/?view_only=715af318a6e04a87a280ea830dd5f10c*)*. Since the dogs included in this study were not used for experimental purposes or medical trials, there are no (inter)national guidelines for the ethical consent of including dogs in animal-assisted therapy research. Following recommendations by other authors in this field [15], we provide a detailed description of the participants, content of the therapy sessions, and measurements below.

### Participants

A total of 71 participants (7 - 16 years) participated in this study. Within each condition, 2 children dropped out before or at the beginning the study, resulting in a final sample of 65 children (*M*_*age*_ 11.24 years; *SD* 2.29 years). Children dropped out for scheduling reasons (*n* =  2), because it was impossible to assign the child to a known therapist (*n* =  1), or because the therapy tasks seemed too difficult after a home visit by the researcher (*n* =  1) or after the first therapy session (*n* =  2). Table 1 shows participants’ characteristics by condition. All children had an official diagnosis set by experts (e.g., pediatrician, a child and adolescent psychiatrist). Parents reported no severe physical, motor, or sensory impairments in their children in a screening questionnaire. All children could understand simple verbal instructions and could express themselves verbally. Most of the children were of Dutch nationality, one was Belgian (Flemish/Dutch-speaking), and one was Dutch/Polish.

**Table 1:** Participant characteristics by condition.

	Number of participants	male: female ratio	Mean age (*SD*)	ASD: DS ratio	Comorbidity
Dog	24	15: 9	10.96 (*2.05*)	11: 13	ADHD (3) Anxiety (1) Hyperthyroidism (1)
Robot dog	21	13: 8	11.38 (*2.51*)	11: 10	FASD and ADHD (1) Anxiety (1) Dyspraxia (1)
Control	20	12: 8	11.45 (*2.40*)	9: 11	ADHD (2) Anxiety (2) Low-impact Hemiplegia (1)
Total	65	40: 25	11.24 (*2.29*)	31: 34	13

*Note:* ASD =  Autism spectrum disorder; FASD =  Fetal alcohol spectrum disorder.

The children with autism were diagnosed with autism “requiring support” (*n* =  8), autism “requiring substantial support” (*n* =  6), Asperger’s syndrome (*n* =  3), Pervasive Developmental Disorder not otherwise specified (*n* =  3), 16p13.11 microduplication with autistic features (*n* =  1), or an autism diagnosis without further specification (*n* =  10). Based on earlier administered IQ tests, parents reported that two children with autism had a mild intellectual disability (50-69), four had a borderline intellectual disability (70-79), seven had below-average IQs (80-89), nine had average IQs (90-109), five had high-average IQs (110-119), and two had IQs well-above average (120 or higher). Of 2 children with autism, the IQ scores were unknown. Most children with DS had a mild intellectual disability (IQ 50-69; *n* =  11), six children had a moderate intellectual disability (IQ 25-49), and four were diagnosed at borderline intellectual disability (IQ 70-79), as reported by their parents. The IQ scores of 13 children with DS were unknown.

The children diagnosed with DS had lower IQ scores on average than the children diagnosed with autism, *χ*2(8, *N* =  65) =  121.77, *p* < .001, Cramer’s *V* = .93, which seems to be in line with population estimates [36]. There was, however, no significant difference in IQ between the three conditions, *χ*2(8, *N* =  65) =  16.63, *p* = .55, and no significant age difference, *F*(2,65) = .30, *p* = .75, and no significant difference in the distribution of males and females, *χ*2(3, *N* =  65) = .03, *p* = .99.

### Procedure

Following ethical approval, the study was conducted in several data collection waves between September 2019 and the April 2022. Parents were recruited through social media and parent organizations for children diagnosed with autism or DS in the Netherlands. After registering on the study website, parents received an information packet about the study, including pictures with pictograms to discuss the study with their child. All parents gave oral and written consent, in addition, the children provided oral consent. Children and parents could withdraw from the study at any time without consequences, and were encouraged to ask any questions they might have.

After giving written consent, parents received a link to an online questionnaire about their child’s social and emotional behavior designed using Qualtrics (https://www.qualtrics.com). The questionnaire contained open-ended and closed-ended questions and could be completed in 10 minutes at the parents’ convenience. The data were stored on a secure disk of the host university. After the first questionnaire was completed, a researcher visited all children at home to explain the study and administer a collaborative task during which parent-child movement synchrony was measured, which is described elsewhere [24]. The home visits were repeated after the 5 therapy sessions or waiting period.

After the first home visit, children in the dog-assisted therapy group were assigned to a licensed therapist (psychologist or disability specialist) associated with this study who worked near their area of residence. The therapists were recruited through the study website before the start of the study. They had all received higher education (applied university level or higher) for supporting children with disabilities or atypical development. In addition, they had received additional training in dog behavior and dog-assisted interventions and operated as an independent therapist for at least several years. The dogs were certified by well-known organizations in the Netherlands. The breed of the dogs varied, although they were often (mixed) retriever breeds (e.g., *Labrador*) or water dogs (e.g., *Poodle*). The therapists indicated in a questionnaire that they complied with the welfare rules of the International Association of Human-Animal Interaction Organizations [2], meaning that they ensured adequate access to water, a limited number of working hours per week and that they were aware of their dog’s stress signals.

Because of the large pool of therapists who had signed up, most children could be assigned to a therapist within a 30-minute travel distance from their home. Parents received the contact details of this therapist and were asked to schedule 5 weekly 45-minute sessions. Therapists were paid per session from the research budget, without any parental involvement. They were asked to structure the sessions according to a predefined protocol (see below). All sessions were held indoors to make the situation comparable to the robot dog condition. After helping their children become acquainted with the dog and the therapist, parents left the intervention room and took a seat in a separate room.

Children in the robot dog condition were asked to visit the host university for five weekly 45-minute sessions of robot-assisted therapy. These sessions were structured in the same way, with the same protocol as the dog-assisted sessions, and were delivered by the first author of this article. Children assigned to the no-treatment control group began with a waiting period. After the post-test and follow-up measurements, these children received one dog-assisted therapy session, paid for from the research budget, by one of the therapists involved in the study, as a gift for their participation.

### Therapy protocol

The therapists followed a predetermined protocol so that all children received comparable therapy sessions. During the therapy, the children playfully practiced their social skills with the help of the dog, for example through emotion recognition and exercises targeting social self-confidence. In addition, social attunement between child and dog was practiced while they completed a (self-built) obstacle course together, with the child taking the lead. This self-built obstacle course was first introduced in the second session and repeated and expanded in sessions 3 to 5. All exercises were based on example exercises from the CTAC (Centro de Terapias Asistidas con Canes) method [37] and an earlier small-scale study [6], and were chosen based on the low level of difficulty to perform, limited use of additional equipment, and suitability for both real dogs and the robot dog.

The robot-assisted therapy used the WowWee CHiP robot dog (WowWee, 2023) with a length of 30, a width of 20, and a height of 18 cm. This robot dog has sensors to respond to the environment and can perform simple commands such as sit or lie down, bark, and ‘dance’. To perform these actions, the robot responds to the child’s voice or touch. Using a Bluetooth connection, CHiP can chase a ball and follow the child around the room when the child wears a wristband and gives the command ‘come here’. CHiP can be rewarded with a pat or a treat given via the app on a connected smartphone or tablet. The robot dog has a (somewhat limited) range of emotions. He can bark, give a ‘kiss’, get angry when pushed, or sad if the connection with the ball is lost. The robot dog will bark, scurry around, or ‘sniff’ on its own at times when no command is given.

Table 2 shows how the therapy sessions were organized. Each session focused on one social-emotional theme. The tasks were exactly similar in the dog and the robot dog condition. Only the command ‘wait and stay’ could not be practiced with the robot dog. Instead, therapist and child had a brief discussion about the weekly theme of cooperation and friendship, in the presence of CHiP.

**Table 2:** Themes and content of the therapy sessions with the dog or robot dog.

Session and theme	Exercises and tasks
1. Getting to know each other	Greeting and introducing each other Recognize feelings of (robot) dog and child Walk together with (robot) dog or tag along Learn to say (robot) dog goodbye
2. Learning to work with a (robot) dog	Commands: Sit, lie down, give paw (dog) or dance (robot dog) Walk around and call (robot) dog to come here What did you think of this lesson?
3. Waiting and working together	Repetition and extension of commands ‘Wait and stay’ (for robot dog: discussion about friendship) Build an obstacle course and complete it with (robot) dog What went well this lesson, what was difficult?
4. Emotions	Acting out and recognizing emotions of the child, therapist and (robot) dog ‘Keep away game ‘(Piggy in the Middle) with the (robot) dog Expand obstacle course and complete it with (robot) dog What went well this lesson, what was difficult?
5. Confidence	Crazy dance with and without the (robot) dog Post football where the therapist deliberately breaks the rules Expand obstacle course and complete it with (robot) dog Saying goodbye and end of sessions

### Measurements – Questionnaire

The online questionnaire was developed especially for this research and first extensively analyzed with respect to internal consistency, scale construction, and reliability by means of a Mokken scale analysis (see below). Parents were asked to rate items describing their child’s social behaviors, by determining how often in the past month their child displayed these behaviors using a five-point Likert scale with the options never, rarely, sometimes, often, and very often [24]. The 66 items were based on five relevant constructs in the literature on social skills and functioning, such as interpersonal synchrony and social attunement [38,39], social cognition and understanding social information [40,41], emotion regulation and impulse control [42], social motivation and showing an interest in others [41], and social anxiety and confidence [43]. The items were worded both positively and negatively. Before the study, six experts who work with the target groups in clinical practice or research, confirmed that they considered the questionnaire suitable for measuring the social strengths and challenges of children with DS or autism.

### Analysis

#### Scale construction.

To confirm the pre-defined scales and assess scale reliability, we carried out a Mokken scale analysis (MSA) on the pretest questionnaire data, according to the stages proposed by [44]. Using the R package Mokken (v 3.0.6), the analysis consisted of three consecutive stages. In the first stage, negatively worded items were recoded, so that for all items a higher score indicated better social skills. We inspected the data for missing data and outliers, searching for notable item response patterns by computing item means. No missing data was present in the pretest data.

During the second stage we assessed scalability by exploring H coefficients for the pre-defined scales and the separate items pertaining to each of these original scales. Scalability coefficients of ≥ .30 were considered satisfactory. If any items were flagged as unsatisfactory, we used the automated item selection procedure (AISP) from the Mokken package (v3.0.6) [45,46]. This procedure supports the identification of items that impair the strength of the scale. More specifically, as suggested by [47], we performed the AISP several times, with incremental values for the cutoff point. That is, we started with a cutoff point of zero, ran the AISP, and subsequently reran the AISP with fixed increments of.05, until the cutoff was.55. No comparison between these steps was conducted. Rather, we compared our outcome pattern of all steps together to the two ‘typical’ outcome patterns (see also [44] for an elaboration and illustration of this procedure). We assumed the monotone homogeneity model and examined the assumptions of local independence and monotonicity accordingly. Concerning local independence, no clear guidelines exist as to how much local non-independence is acceptable in relation to the number of items [48]. In case of local non-independence, the scalability coefficient could be somewhat overestimated. As such, we decided to accept any remaining local non-independence, if the other indices pointed towards an acceptable scale in combination with a good substantive interpretability. Finally, we examined test reliability per scale by estimating Guttman lambda-2. Based on the above outcomes we decided on removing certain items, and retest the remaining scale until the scale seemed satisfactory in terms of a combination of type of outcome pattern during the AISP, scalability, reliability and assumptions (see below). Importantly, removal of items was discussed between the first and second author to assess whether this removal was also justified based on substantive considerations. A detailed overview of results of the final scales and items is presented in Tables S1 and S2 in the online supporting information.

#### Social confidence.

The MSA procedure resulted in a final Social Confidence Scale of 9 items with a satisfactory scalability coefficient of 0.37, 95% CI [.25,.48] (example item “My child is confident in interacting with others”). All individual scalability items were above (or very close to).30, one item showed a minor violation of monotonicity. Around 18% of item pairs showed signs of local dependence. The 10^th^ item of the original scale (“My child seeks proximity to caregivers”) was removed due to a notable item response pattern, signs of non-monotonicity and local nonindependence, and unscalability during the AISP procedure. In terms of content, this specific item was considered less appropriate to probe social confidence as it may more reflect characteristics of the parent-child attachment.

#### Attunement (conversational and emotional).

Based on the MSA procedure, we decided to create two separate scales, a Conversational Attunement Scale (8 items; example item “My child talks out of turn in a conversation”) and an Emotional Attunement Scale (6 items; example item “My child comforts others in times of sadness or pain”). Both scales showed satisfactory scalability (H-coefficient of.39, 95% CI [.27,.49] and.44, 95% CI [.30,.55] respectively). The Emotional Attunement Scale showed local nonindependence for one (7%) item pair. Concerning the Conversational Attunement Scale, one item (“My child takes longer to respond”) showed many signs of un(satisfactory) scalability in early phases of the AISP. Content-wise, this item probably better reflects processing speed than attunement, and was therefore removed from the scale.

#### Emotion regulation.

The final Emotion Regulation Scale consisted of 15 items (H-coefficient of.42, 95% CI [.30,.51], example item “My child explodes seemingly out of nowhere”), with a local nonindependence for one (1%) of the item pairs, and some minor violation of monotonicity for 2 items. Three of the original items were removed due to unsatisfactory individual scalability. One example of such an item was “My child shows pleasure”, which differed from the other items because this may reflect the sharing of emotions rather than the child’s control of his or her emotions.

#### Social cognition.

The final Social Cognition Scale consisted of 8 items (H-coefficient of.36, 95% CI [.23,.47], example item “My child does not understand the gist of a conversation”), with a local nonindependence for one (3%) item pair. Three items from the original scale were removed as these were flagged as unscalable or as belonging to a separate subscale. These items (e.g., “My child understands other people’s actions”) likely show a great deal of individual variation, depending not only on the child’s social cognition, but also on the intellectual capacity and nature of other people’s actions. This likely resulted in inconsistency within this scale, justifying its removal.

#### Social motivation.

The final Social Motivation Scale showed acceptable scalability (10 items; H-coefficient of.36, 95% CI [.24,.45], example item “My child does not make contact with others on their own”), with a local nonindependence for three (7%) item pairs. Two of the original items seemed less fitting, and were allocated to a separate subscale during the initial AISP. These items (e.g., “My child reacts to everything”) seemed to relate more to (problems with) inhibition/control and less to motivation to interact with others and were therefore removed from the final scale.

The reliability analysis for the final scales showed sufficient reliability for group-level studies (all Guttman lambda-2 ≥ .80). To create total scale scores we computed the mean of the items that constituted the final scales based on the Mokken Scale Analysis (MSA). These scores were used for all subsequent analyses. Descriptive analyses were conducted in SPSS (version 27), while R (version 4.2.0) was used for all other analyses.

#### Multi-level repeated measures analysis.

To examine the effect of the real and robot dog intervention condition, we carried out two-level multilevel models, with repeated measures nested in participants, for each scale separately. At this time, we included both the children with autism and DS in the same analysis, considering that we explore the data at a more individual level below. An overview of the model building is presented in Table 3. Time was treated as a continuous variable (coded as 0, 1, 2), in order to maximize the ratio of information to parameters to be estimated. The intervention was dummy coded, with the control condition as the reference category. The intervention effect was entered in the final model (model 5, see Table 3) as the time*intervention interaction effect. The random slope of time (tested in model 4), was only included in the final model if statistically significant. The final model was compared to the previous model (either model 3 or 4) by means of a deviance test (ANOVA function within lme4 package).

**Table 3:** Multi-level model building.

Model	Fixed effects	Random effects
1	Intercept	Intercept
2	Intercept, time (level 1)	Intercept
3	Intercept, time (level 1), condition^a^ (level 2)	Intercept
4	Intercept, time (level 1), condition^a^ (level 2)	Intercept, slope of time
5	Intercept, time (level 1), condition^a^ (level 2), interaction of condition * time	Intercept, slope of time^b^

*Note.* ^a^ Condition was entered as dummy variable with the control condition as reference category. ^b^ Only if significant in previous model.

All multi-level analyses were conducted using the R packages lme4 (version 1.1-32), lmerTest (for obtaining *p*-values; version 3.1-3) and performance (for obtaining intra class coefficients [ICC]; version 0.10.5). Concerning effect size of the intervention effect, we computed the 95% confidence interval surrounding the estimated effect, and interpreted the clinical relevance of the resulting effect range. Finally, we checked for violations of normality of residuals at the within and between levels, and of homoscedasticity. Any violations were only minor (see Figs S1 and S2 in the online supporting information) and therefore we decided to follow-through with the multilevel modeling (see [49]).

#### Reliable change index.

To gain insight into the intervention effect per scale on an individual level, we computed the reliable change index (RCI) according to the equation of [50]. To account for differences in variance between time-points we computed the standard error of the difference according to [51]. Reliable change scores that exceeded ±  1.96 (95% confidence interval) were considered to represent reliable change, or in other words, change that was not due to chance or measurement error and therefore might indicate clinical relevance [52]. Each child’s RCI was computed for the change from pretest to posttest, and from pretest to follow-up.

To compare the mean RCIs between the conditions we conducted Monte Carlo permutation one-way ANOVA’s (wPerm, Version 1.0.1). This non-parametric alternative was chosen because of a lower power when using standard parametric tests (.40; G * power, alpha = .05, medium effect size). That is, a nonparametric test poses less strict assumptions on the distribution of data. The distribution of the test statistic is based on the actual sample properties. As a result, parameter estimates and p-values are relatively unbiased [53]. Post hoc comparisons between conditions were examined through pairwise permutation tests (R companion, Version 2.4.0), with a false discovery rate correction to avoid inflation of Type I error. Post hoc comparisons were carried out for all ANOVAs that resulted in a statistically significant omnibus test. Since significant post hoc comparisons can occur in situations where the omnibus test is non-significant [54], we conducted post hoc comparisons for all omnibus tests with a medium-sized effect.

Lastly, we did an explorative analysis of the highest (90^th^ percentile) and lowest (10^th^ percentile) RCIs per scale by examining the percentages of participants with the highest and lowest RCIs for each condition and for the diagnosis, sex, and IQ score categories.

## Results

### Descriptive analysis

**Fig 1:**
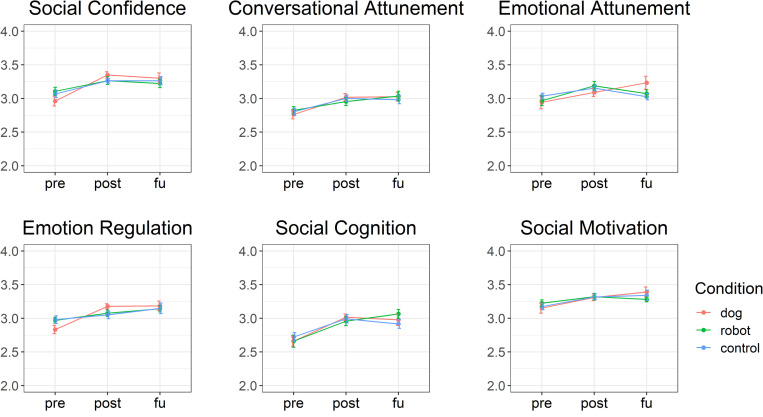
Development of Social Skills from Pre-test to Follow-up per Experimental Condition.

Table 4 presents an overview of means and standard deviation for the total sample, as well as per time-point and intervention condition. Overall, there was an increase in mean social skills from pretest to posttest, for all scales and for all conditions (see Fig 1). Change was less apparent from post-test to follow-up.

**Table 4:** Means and standard deviations of social skill scales.

Scale	Pre-test		Post test		Follow-up	
	*M*	*SD*	*M*	*SD*	*M*	*SD*
Social Confidence						
Dog	2.96	.36	3.35	.23	3.30	.33
Robot	3.11	.28	3.27	.26	3.22	.23
Control	3.07	.23	3.26	.16	3.26	.24
Conversational Attunement						
Dog	2.76	.32	3.02	.27	3.03	.27
Robot	2.82	.25	2.95	.27	3.04	.28
Control	2.80	.23	3.00	.22	2.98	.24
Emotional Attunement						
Dog	2.94	.48	3.09	.29	3.23	.41
Robot	2.97	.33	3.19	.30	3.07	.21
Control	3.03	.19	3.15	.20	3.03	.19
Emotion Regulation						
Dog	2.83	.30	3.18	.19	3.18	.30
Robot	2.97	.21	3.08	.21	3.14	.18
Control	2.98	.24	3.05	.25	3.15	.32
Social Cognition						
Dog	2.66	.38	3.02	.23	2.98	.35
Robot	2.66	.42	2.96	.31	3.07	.25
Control	2.72	.29	2.99	.28	2.92	.26
Social Motivation						
Dog	3.15	.38	3.31	.24	3.39	.32
Robot	3.22	.23	3.32	.23	3.28	.14
Control	3.17	.20	3.32	.19	3.34	.30

### Multilevel analyses

All missing data (pretest 1.5%, posttest 8%, follow-up 24%) concerned participant non-response. Multi-level modeling handles missing data well because it estimates parameters based on the hierarchical structure, allowing incomplete cases to contribute partial information, and accounts for variability at different levels. Additionally, we employed maximum likelihood estimation, which is robust to missing data and unbalanced data [49].

The main effect of time (Model 2) was significant and positive for most scales, indicating that on a group-level most social skills improved over time. The random slope of time (Model 4) was non-significant for most scales and resulted in a boundary fit for the Conversational Attunement and the Social Cognition scales. Concerning the boundary fits, variance around the time slope was estimated to be zero. Therefore, all final models (Model 5) were fit without a random slope of time.

The results of the final model per scale are presented in Table 5. A significant time*condition effect was found for the dog condition for the Emotional Attunement scale. In other words, children who received dog assisted therapy improved more in terms of Emotional Attunement as compared to children in the control and robot dog condition. It should be noted though that the deviance test indicated that the final model (Model 5) did not fit the data significantly better than the previous one (Model 3). For the Emotion Regulation scale, the final model revealed a borderline significant interaction effect for the dog condition. Again, model fit did not improve significantly from Model 3 to the final model as indicated by a non-significant deviance test.

**Table 5:** Statistics for multilevel models for the final models per social skills scale.

	Social Confidence	Conversational Attunement	Emotional Attunement	Emotion Regulation	Social Cognition	Social Motivation
*Fixed effects*	*B (SE)* 95% CI [LL, UL]					
Intercept	3.07 (0.17)^***^ [2.81, 3.33]	2.77 (0.15)^***^ [2.48, 3.05]	2.99 (0.13)^***^ [2.74, 3.25]	2.92 (0.13)^***^ [2.68, 3.20]	2.70 (0.12)^***^ [2.47, 2.94]	3.12 (0.12)^***^ [2.88, 3.37]
*Within child level*						
Time	0.11 (0.05)^*^ [0.02, 0.21]	0.10 (0.05)^*^ [0.01, 0.19]	0.006 (0.06) [-0.10, 0.13]	0.09 (0.04)^*^ [[0.00, 0.18]	0.11 (0.06)^†^ [-0.01, 0.23]	0.09 (0.05)^*^ [0.01, 0.18]
*Between child level*						
Condition Dog^a^	-0.04 (0.18) [-0.04, 0.33]	0.03 (0.20) [-0.33, 0.43]	0.01 (0.18) [-0.36, 0.35]	-0.02 (0.18) [-0.40, 0.31]	0.09 (0.16) [-0.22, 0.39]	0.07 (0.16) [-0.26, 0.39]
Condition Robot dog^a^	0.01 (0.19) [-0.32, 0.40]	0.18 (0.20) [-.21, 0.60]	0.01 (0.19) [-0.36, 0.33]	0.04 (0.18) [-0.30, 0.43]	-0.03 (0.17) [-0.35, 0.30]	0.12 (0.17) [-0.22, 0.46]
Condition Dog ^*^ Time	0.09 (0.07) [-0.04, 0.23]	0.05 (0.06) [-0.08, 0.16]	0.15 (0.08)^*^ [-0.01, 0.30]	0.11 (0.06)^†^ [-0.01, 0.23]	0.07 (0.08) [-0.11, 0.22]	0.04 (0.06) [-0.08, 0.18]
Condition Robot dog^*^ Time	-0.05 (0.07) [-0.18, 0.08]	0.02 (0.07) [-0.11, 0.14]	0.06 (0.08) [-0.10, 0.21]	0.002 (0.06) [-0.12, 0.12]	0.10 (0.08) [-0.05, 0.26]	-0.06 (0.06) [-0.19, 0.07]
*Random effects*	*Variance*					
$\sigma_{}^{2}$ (within)	.08	.35	.11	.07	.11	.07
$\sigma_{}^{2}$ (intercept)	.30	.35	.27	.28	.19	.23
$\sigma_{}^{2}$ (slope)	n/a	n/a	n/a	n/a	n/a	n/a
Deviance	207.34	207.75	231.70	179.00	219.45	174.83
∆χ^2^(df)^b^	4.47 (2)	0.56 (2)	4.19 (2)	4.38 (2)	1.65 (2)	2.62 (2)

*Note.* Standard errors within brackets.

^a^Dummy variable with the control condition as reference category.

^b^Comparison with Model 4 (in case of significant slope of time) or Model 3.

† *p*

< .10.

* *p* < .05.

** *p* < .01.

*** *p* < .001

### Reliable change index (RCI)

**Fig 2:**
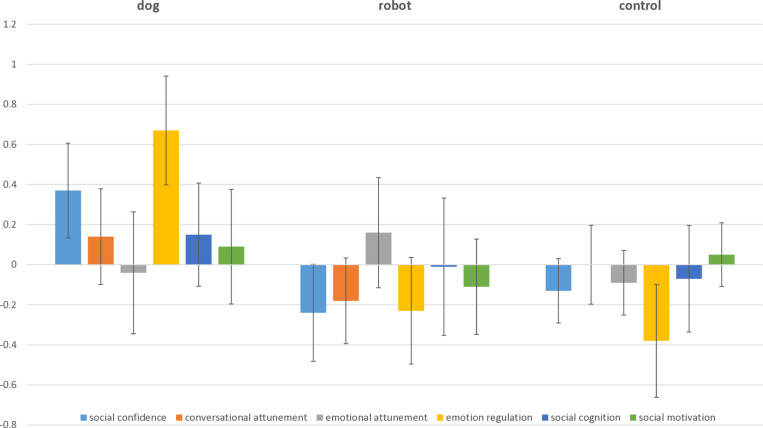
Mean Reliable Change Index by Condition (Pre−test − Post−test Differences).

The amount of missing data for this analysis was the same as for the multi-level analysis. We used pairwise deletion: RCIs were only computed when data on both measurement occasions was available. Table 6 shows the means and standard deviations of the Reliable Change Index (RCI) from pretest to posttest and from pretest to follow-up. Concerning the reliable change from pre- to posttest, we found a non-significant, yet medium-sized omnibus effect for the social confidence scale (F (2, 63) =  2.36, *p* = .10, η^2^ = .07). Post hoc comparisons showed no substantive differences in reliable change between the conditions. For the Emotion Regulation scale, we found a significant and medium-sized effect of condition (F (2, 63) =  4.48, *p* = .02, η^2^ = .13). Post hoc comparisons revealed that children in the dog condition (M_RCI_ = .67) showed a significantly higher reliable change as compared to the robot (M_RCI_ =  -.24; 95% CI of mean difference [-1.76, -.13]; Cohen’s *d* = .36) and the control (M_RCI_ =  -.38; 95% CI of mean difference [-1,84, -.25]; Cohen’s *d* = .33) condition (see Fig 2).

**Table 6:** Means, standard deviations and range of reliable change index.

Scale	Pre−test–Post−test		Pre−test–Follow−up	
	*M (SD)*	*Range*	*M (SD)*	*Range*
Social Confidence				
Dog	0.37 (1.16)	−1.28–3.53	0.19 (1.51)	−2.61–2.79
Robot	−0.24 (1.11)	−2.48–2.63	−0.43 (1.02)	−1.71–1.29
Control	−0.13 (0.72)	−1.28–1.13	−0.27 (1.01)	−1.71–2.19
Conversational Attunement				
Dog	0.14 (1.17)	−1.84–2.43	0.36 (1.07)	−1.00–2.35
Robot	−0.18 (0.98)	−1.51–1.78	0.23 (0.90)	−2.22–1.74
Control	0.01 (0.88)	−1.18–2.11	0.03 (0.81)	−1.61–1.13
Emotional Attunement				
Dog	−0.04 (1.49)	−3.40–2.58	0.44 (1.86)	−3.05–4.21
Robot	0.16 (1.26)	−2.55–2.15	−0.14 (1.04)	−2.62–1.65
Control	−0.09 (0.72)	−1.27–1.30	−0.49 (0.70)	−1.34–1.22
Emotion Regulation				
Dog	0.67 (1.33)	−1.68–3.70	0.55 (1.81)	−3.85–3.31
Robot	−0.23 (1.22)	−2.45–1.91	−0.03 (0.89)	−1.63–1.33
Control	−0.38 (1,26)	−2.45–1.91	−0.19 (1,53)	−2.37–3.31
Social Cognition				
Dog	0.15 (1.26)	−1.88–3.68	0.14 (1.79)	−1.99–4.63
Robot	−0.01 (1.57)	−1.88–3.68	0.47 (1.58)	−1.62–3.53
Control	−0.07 (1.19)	−2.92–1.94	−0.35 (1.14)	−1.62–2.43
Social Motivation				
Dog	0.09 (1.40)	−2.52–3.55	0.30 (1.73)	−3.44–4.06
Robot	−0.11 (1.09)	−1.91–2.03	−0.50 (0.80)	−1.56–0.94
Control	0.05 (0.71)	−1.30–1.12	−0.14 (1.25)	−2.81–2.50

Concerning reliable change from pretest to follow-up, we found a non-significant, yet medium-sized omnibus effect for the Emotional Attunement scale (F (2, 48) =  2.13, *p* = .13, η^2^ = .09). Post hoc comparisons showed that the reliable change was higher in the dog (M_RCI_ = .44) than in the control condition (M_RCI_ =  -.48; 95% CI of mean difference [-2.01,.42]). This difference, though non-significant, was of a medium effect size (Cohen’s *d* = .30).

When looking at the RCI on a more individual level, grouping the research participants per scale by their RCI, the highest RCIs (in the 90th percentile) are found in the dog−assisted therapy group (see Table 7). Yet, for the Emotional Attunement and Social Motivation scales we also see high RCIs in the robot−assisted group. Males with autism have the highest reliable change on 3 of the 6 questionnaire scales and girls with DS have the highest reliable change on 2 of the 6 scales. In general, children with the lowest IQ scores seem to have the highest RCIs. Children assigned to the control group, females with autism and children with the highest IQ scores do not reach the 90^th^ percentile of RCIs on any of the scales.

**Table 7:** Percentages of participants with a high RCI (90th percentile) by category.

	Sample %	Social Conf. (*n* = 6)	Convers. Attunement (*n* = 6)	Emotional Attunement (*n* = 7)	Emotion Reg. (*n* = 8)	Social Cognition (*n* = 7)	Social Motiv. (*n* = 7)
Dog	*37*	**83**	**67**	**57**	**63**	43	**57**
Robot	*32*	17	17	**43**	25	29	**43**
Control	*31*	0	17	0	13	29	0
male ASD	*33*	33	**67**	**43**	25	**43**	29
male DS	*30*	**50**	0	14	25	14	14
female ASD	*14*	17	0	14	13	14	14
female DS	*23*	0	**33**	29	**38**	29	**43**
low IQ	*42*	50	33	**71**	50	**57**	43
average IQ	*23*	17	**33**	29	25	29	**43**
high IQ	*11*	0	17	0	13	14	0
IQ unknown	*23*	**33**	17	0	13	0	14
Highest RCI	*n/a*	dog, male ASS, average IQ	dog, female DS, low IQ	dog, male ASS, average IQ	dog, girl DS, low IQ	robot/dog, girl DS, low IQ	dog, male ASS, average IQ

*Note:* ASD =  Autism spectrum disorder. All percentages are rounded. The first column displays the percentage of the sample that falls into each category (e.g., 37% of the sample was assigned to the dog−assisted therapy condition). The other columns display the percentages of participants with a high RCI (90th percentile) that fall into each category (e.g., of the participants with the highest RCI on the Social Confidence scale, 83% were in the dog−assisted therapy condition, which is higher than the sample percentage). Numbers in bold indicate that the percentage within a specific category is at least 10 percentage points higher than the sample percentage.

In contrast, the lowest RCIs (in the 10th percentile) are more common in the robot−assisted therapy group (see Table 8). Only for the Emotional Attunement and Social Motivation scales do we also see low RCIs in the dog−assisted group. Males with DS have the lowest RCIs on 4 of the 6 scales. Notably, children with an unknown IQ score have the lowest RCIs on 4 of the 6 scales. In general, children assigned to the control group, males with autism and children with average and high IQ scores do not have RCIs in the 10^th^ percentile on any of the scales.

**Table 8:** Percentages of participants with a low RCI (10th percentile) by category.

	Sample %	Social Conf. (*n* = 9)	Convers. Attunement (*n* = 11)	Emotional Attunement (*n* = 7)	Emotion Regulation (*n* = 7)	Social Cognition (*n* = 7)	Social Motivation (*n* = 11)
Dog	*37*	33	36	**71**	14	14	**55**
Robot	*32*	**56**	**55**	14	29	**71**	36
Control	*31*	11	9	14	**57**	14	9
male ASD	*33*	22	27	14	29	29	18
male DS	*30*	22	**45**	29	**57**	**43**	**45**
female ASD	*14*	11	9	**29**	0	0	18
female DS	*23*	**44**	18	29	14	29	18
low IQ	*42*	33	27	43	29	**57**	45
average IQ	*23*	22	18	14	14	29	9
high IQ	*11*	0	0	14	0	0	9
IQ unknown	*23*	**44**	**55**	29	**57**	14	**36**
lowest RCI	*n/a*	robot, female DS, low IQ	dog, male DS/ASD, low/unknown IQ	dog, male DS, IQ unknown	control/robot, male ASD/DS, IQ unknown	control, male DS, low IQ	dog, male ASD, low IQ

Note: ASD =  Autism spectrum disorder. All percentages are rounded. The first column displays the percentage of the sample that falls into each category (e.g., 37% of the sample was assigned to the dog−assisted therapy condition). The other columns display the percentages of participants with a low RCI (10th percentile) that fall into each category (e.g., of the participants with the lowest RCI on the Social Confidence scale, 33% were in the dog−assisted therapy condition, which is below the sample percentage). Numbers in bold indicate that the percentage within a specific category is at least 10 percentage points higher than the sample percentage.

## Discussion

This study compared children (aged 7 to 16 years) with autism or Down syndrome (DS) who received five sessions dog−assisted therapy with an active control group who received five sessions robot−assisted therapy and a no−treatment control group. The robot dog was capable of performing autonomous behavior, obeying commands, showing emotions, and following children around the room, but also limited in its ability to engage in true reciprocal interactions. We assessed children’s social and emotional skills (i.e., social confidence, conversational and emotional attunement, emotion regulation, social cognition and motivation) before and after the therapy sessions or waiting period using parental questionnaires. We also included a follow−up measurement after 4−6 weeks. Our main research question was whether there would be a difference between the group receiving dog−assisted therapy (*n* =  24), the group receiving robot−assisted therapy (*n* =  21), and the no−treatment control group (*n* =  20). However, because children with autism and DS can be so diverse on many characteristics [24], and therapists in the field tailor their interventions to specific children, we also looked at the impact on an individual level.

Our results indicate an increase in mean social skills from pretest to posttest for all questionnaire scales. Change from post−test to follow−up was less apparent. Multilevel analyses showed that children who received dog−assisted therapy improved significantly more in terms of emotional attunement than children in the control condition. For the Emotion Regulation scale, we found a borderline significant interaction effect for the dog−assisted therapy condition. For all other scales, no significant differences between the dog and control condition or between the robot dog and control condition were found. The hypothesized positive effect of therapy with a real dog on children’s social skills was found only for emotional attunement and, to some extent, emotion regulation. This also means that for some children, such as those with allergies or a fear of dogs, or in settings where the assistance of dogs may not be feasible, therapy with a robot dog could be a promising alternative.

At the same time, individual results show that *some* children’s conversational attunement, social confidence, social cognition and social motivation clearly benefit from dog−assisted therapy. The Reliable Change Index (RCI) was generally more positive for children who received dog−assisted therapy, across all questionnaire scales. Especially when looking at the 90^th^ percentile, most of the highest RCIs were found in the dog−assisted therapy group. In contrast, most of the lowest RCIs—within the 10^th^ percentile—were found in the robot−assisted group. In general, children with the lowest IQ scores appeared to have the highest RCIs. Children assigned to the no−treatment control group, females with autism, and children with the highest IQ scores did not reach the 90^th^ percentile of RCIs on any of the questionnaire scales. For the Emotional Attunement and Social Motivation scales, it is also interesting to note that both high and low RCIs were found more frequently in the dog−assisted therapy group, indicating that some children made considerable progress while others did not. In summary, this study’s findings are complex. Group results do not *always* favor the dog−assisted therapy group; individual results more often do.

It is particularly noteworthy that we do find a group effect in favor of dog−assisted therapy for the questionnaire scales Emotional Attunement and Emotion Regulation. Perhaps interactions with a (real) dog primarily affect children’s feelings and how they interact and cope with them. Indeed, literature reviews show more positive emotions after receiving dog−assisted therapy for both children with intellectual disabilities, such as Down syndrome [23] and children with autism [17]. A stress−reducing effect of interacting with animals, as mentioned by several authors, may be a possible mechanism to explain this effect [4,55,56]. For example, a study by [57], in which (adult) pet owners were asked to record their feelings and whether they were in the presence of their pet (dog or cat) at random times for five consecutive days, showed that having a companion animal helps to maintain positive feelings when under stress. The authors called this a buffering effect because when no stress was reported, no effect on positive emotions was found. Although this study was with adult pet owners [57], this may be an indication that the mechanism of animal−assisted therapy could be somewhat more complex. Possibly an effect of stress reduction and emotion regulation occurs first, after which there is room for other positive effects on social behavior. However, the current study shows that these additional effects may depend on whether the intervention is maintained over a longer period of time, because our 4−6 week follow−up measurement showed no additional effects. On the contrary, the effect on emotion regulation and attunement decreased slightly from posttest to follow−up.

Although this is the first study with a controlled design and a series of five similar therapy sessions with either a dog or robot dog, the use of robots to promote social skills has been investigated in other studies. For example, [58] and [59] investigated joint attention in children with autism using a humanoid robot. An earlier study [30] examined differences in typically developing children’s responses to real and robot dogs. They showed that although children generally viewed the robot dog as an object, they still interacted with the robot dog as they would with a real dog by talking to it, giving commands, and playing. In a more recent study [33], 13 adults and 10 children with autism were exposed to an interaction with a researcher only, a researcher and a real dog, and a researcher and a robot dog in a counterbalanced design. The robot dog was comparable to the robot dog used in the current study. Participating adults showed more adaptive social behavior and communication skills in both the dog and robot conditions compared to the researcher−only condition. For the children, however, positive results were found only in the real dog condition. Notably, only the real dog had a positive effect on cardiac autonomic function in both children and adults with autism. Thus, while the results regarding the social effects were more complex, the physiological results of [33] were more pronounced in favor of the (real) dog.

Our own exploratory analyses using the RCIs show that also in this study, there are indeed children who benefit more from therapy with a (real) dog. While it is common in many animal−assisted therapy studies, as well as in other intervention studies, to assume a universal effect of interventions [5], we believe our results warrant that a more individualized picture could be useful to the field. Individual differences such as those found in this study may also explain the mixed results found in previous group−based studies of animal−assisted interventions [15]. At the same time, the more individualized approach that we take at the end of the results section is not a silver bullet either. We examined the RCIs in relation to condition, participant gender, diagnosis, and IQ category, but the participating children likely differed on many more characteristics, such as their language development, type of education they receive, whether they have experience or affinity with animals or robots, and the severity of their diagnosis. This raises the question of whether we can ever get a complete and clear picture of which intervention works for whom. The more we divide our sample into smaller groups with specific characteristics, the smaller the groups we are comparing will be. As a result, exploratory analyses become one of the few, if not the only, way to study these differential effects if we were to follow a traditional component−dominant approach. An interesting alternative would be to examine whether characteristics of the interaction dynamics between child and (robot) dog are related to children’s progress after the intervention. In recent years, this complexity approach has been increasingly applied to the study of psychopathology. Authors taking this approach view psychopathology as a pattern of self−organizing interactions between people and their environment (e.g., [60]). Applied to the study of animal−assisted interventions, focusing on the human−animal interaction dynamics, rather than their individual characteristics, may help to explain differential effects and contribute to a more individualized view of animal−assisted therapy.

Our findings should be considered in light of some limitations. To make the comparison between conditions as pure as possible, we designed this study so that the therapists followed similar protocols for the dog and robot dog conditions. However, it is not inconceivable that the child−dog interactions between activities were different from the interactions children had with the robot dog. Differences between the two conditions may therefore be due not only to the protocolled interactions, but possibly also to the informal interactions. It should also be mentioned that there may have been a different degree of novelty between the conditions. In this regard, Marino [25] has argued that animal−assisted therapy is susceptible to novelty effects because the opportunity to interact with animals is not common in therapy contexts. Authors have therefore recommended using control groups with a different novel and exciting stimulus, as we attempted to do in this study [5,20,25]. Yet, it could be argued that the robot dog was newer and more interesting to the children, thereby influencing the results. Moreover, although the children in the dog−assisted therapy group were supported by certified therapists and dogs, not all children were accompanied by the same dog and therapist, due to the sample size and for practical reasons. Given the likely importance of how interactions unfold in the child−therapist−dog triangle, this is an important point to consider.

A second point is the use of our social skills questionnaire. We used a new questionnaire that we felt was more appropriate than standard clinical practice questionnaires, which are often specifically designed for screening or diagnostic purposes, and often use only 3 response options [6]. At the same time, the use of a new questionnaire, even though it was extensively researched using a Mokken scale analysis, means that little is known about its psychometric properties. In line with this, regarding the reliable change index (RCI), there is no consensus in the literature on which test reliability should be used. We used internal consistency based on our own sample [52,61]. Similarly, the cut−off point for the RCI in the literature is somewhat arbitrary. We chose a cut−off point of 95%, which can be considered conservative and may underestimate the true change, but is often used in clinical practice.

This study was conducted during and around the Global Covid 19 Pandemic, where the robot dog condition was administered after the real dog condition, which affected the assignment of children to conditions and may have had unintended effects on the results. Follow−up research will hopefully not be plagued by this challenge and can explore what robot dog therapy might offer children in the longer term. New research should also look critically at the timing of the follow−up measurement (s). Our study’s follow−up took place after 4 to 6 weeks, which means that we have no insight into the longer−term effects of the dog− or robot−assisted therapy, a limitation that other authors in the field of animal−assisted interventions have commented on [5]. Finally, our research could have benefited from detailed observations of both the children’s real−life social behavior before and after the intervention and their behavior *during* therapy sessions with the (robot) dog. For example, did the children who interacted more actively with the (robot) dog progress more in their social−emotional skills?

## Conclusions

This study combined a randomized controlled trial of dog−assisted therapy for children with Down syndrome or autism with a more individualized analysis. Our multilevel analysis showed that not all group outcomes favored the dog−assisted therapy condition over the robot dog condition and the no−treatment control group. That is, the hypothesized positive effect of therapy with a real dog on children’s social and emotional skills was found for emotional attunement and, to some extent, emotion regulation. At the same time, the Reliable Change Index was generally more positive for the children who received dog−assisted therapy, and for all questionnaire scales. This indicates that the children who improved the *most* were more often assigned to the condition with a real dog, rather than the robot dog or the no−treatment control group. While these results yield a more complete picture of the effect of animal−assisted therapy, the questions raised by this study provide an interesting avenue for further research.

## Supporting information

S1 Dataset Anonymized participant background information and questionnaire scale scores at pre−test, post−test, and follow−up.
(XLSX)

S1 Table Constructs, final scale description, and items of the questionnaire on social−emotional skills.
(DOCX)

S2 Table Results of Evaluation of Assumptions, Scalability− and Reliability of the Mokken Scale Analysis.
(DOCX)

S1 Fig Scatterplot of Fitted Social Skill Scores versus Residuals for the Final Model.
(TIF)

S2 Fig Q−Q plots of Within (left) and Between (right)−level residuals for the Final Model.
(DOCX)
