# Supplementary material for: A randomized controlled trial of the effects of dog-assisted versus robot dog-assisted therapy for children with autism or Down syndrome
Source: PLoS One. 2025 Mar 19;20(3):e0319939. doi: 10.1371/journal.pone.0319939 (PMC11922239; doi:10.1371/journal.pone.0319939)
Supplement: S1 Table — (DOCX) [file pone.0319939.s002.docx]

**A Randomized Controlled Trial of the Effects of Dog-Assisted and Robot Dog-Assisted Therapy for Children with Autism Spectrum Disorder or Down Syndrome**

**Supporting information**

Table S1. Constructs, final scale description, and items of the questionnaire on social-emotional skills.

| **(Original) Construct** | **Final Scale** | **Description** | **Final items (positively or negatively phrased)** |
| --- | --- | --- | --- |
| Attunement | Emotional attunement | Putting yourself in the shoes of others, considering emotions and interacting in an emotionally appropriate manner with peers (Salovey & Mayer, 1990). | - My child comforts others in times of sadness or pain - My child offers help to others - My child plays appropriately with peers - My child focuses his/her attention where others focus their attention - My child is good at explaining to others what he/she means - My child lives in a world of their own |
|  | Conversational attunement | Coordination in conversations, reciprocity, considering conversation techniques and listening to others. | - My child makes thoughtless comments - My child talks out of turn in a conversation - My child comes too close to others (in their personal space) - My child talks over other people - My child is a good listener - My child takes things away when someone else is busy with them - My child seems to be on a different wavelength than others - My child talks about topics that only concern him/her |
| Social motivation/collaboration | Social motivation | Seeking contact (“social approach motive”, Coplan et al., 2004). Forming affective and friendly relationships with peers and acting prosocially within this relationship (collaboration, sharing). | - My child does not look for comfort in sadness/pain - My child prefers to be alone - My child does not make contact with others on their own - My child does not participate when asked - My child shares things with others - My child responds to requests - My child spontaneously tells something - My child is interested in other children/young people - My child collaborates with others - My child has difficulty making friends |
| Social confidence/fear in (new) social situations without caregivers | Social confidence | Separation anxiety, fear of social situations with exposure to unfamiliar people” (Stein & Stein, 2008). Self-confidence and resilience, standing up for yourself when necessary (Boelhouwer, 2013). | - My child clings to adults - My child easily separates from caregivers when saying goodbye - My child panics in new situations - My child is tense in social situations - My child is much more restless in social situations than when they are alone - My child makes plans to do things with others - My child stands up for himself when necessary - My child stands up for others when necessary - My child is confident in interacting with others |
| Emotion regulation and frustration, emotion communication | Emotion regulation | Recognizing, acknowledging and managing emotional frustrations, communication about this, and conflict management | - My child gets angry easily - My child stays angry for a long time - My child is too sensitive - My child is sad - My child explodes seemingly out of nowhere - My child is in a good mood - My child is emotionally distant - My child can quickly change moods - My child must always get their way - My child is fussy about little things - My child is persistent - My child finds it difficult to control himself - My child gets frustrated when they cannot explain something - My child gets upset in situations with a lot of stimuli - My child cannot deal with conflict |
| Sociale cognition and understanding of others | Social cognition | “Understand the meaning of social information and interactions with others (Gallese, Keysers & Rizzolatti, 2004). Making yourself understood, contribute to the understanding of others. | - My child does not understand the gist of a conversation - My child does not understand jokes - My child has difficulty following the flow of a conversation - My child understands how events are related (cause/effect) - My child talks about things that are irrelevant - My child has difficulty answering questions clearly - My child gives illogical reasons for what they do - My child can clearly express what they mean |
