# Supplementary material for: A randomized controlled trial of the effects of dog-assisted versus robot dog-assisted therapy for children with autism or Down syndrome
Source: PLoS One. 2025 Mar 19;20(3):e0319939. doi: 10.1371/journal.pone.0319939 (PMC11922239; doi:10.1371/journal.pone.0319939)
Supplement: S2 Table — (DOCX) [file pone.0319939.s003.docx]

**A Randomized Controlled Trial of the Effects of Dog-Assisted and Robot Dog-Assisted Therapy for Children with Autism Spectrum Disorder or Down Syndrome**

**Supporting information**

Table S2. Results of Evaluation of Assumptions, Scalability- and Reliability of the Mokken Scale Analysis.

| **Final Scale** | **Scalability (H-coëfficient)** | | **Reliability (alpha)** | **Monotonicity** | **Local Dependence** |
| --- | --- | --- | --- | --- | --- |
| Social confidence | My child clings to adults | .29 | .81 | Some deviation from monotonicity for item ‘My child easily separates from caregivers when saying goodbye’ | Between ‘My child panics in new situations’ and most of the other items  Between ‘My child clings to adults’ and ‘My child is tense in social situations’ |
|  | My child easily separates from caregivers when saying goodbye | .35 |  |  |  |
|  | My child panics in new situations | .50 |  |  |  |
|  | My child is tense in social situations | .42 |  |  |  |
|  | My child is much more restless in social situations than when they are alone | .39 |  |  |  |
|  | My child makes plans to do things with others | .32 |  |  |  |
|  | My child stands up for himself when necessary | .33 |  |  |  |
|  | My child stands up for others when necessary | .29 |  |  |  |
|  | My child is confident in interacting with others | .45 |  |  |  |
| Conversational attunement | My child makes thoughtless comments | .49 | .80 | No obvious violations | No local dependence |
|  | My child talks out of turn in a conversation | .50 |  |  |  |
|  | My child comes too close to others (in their personal space) | .38 |  |  |  |
|  | My child talks over other people | .46 |  |  |  |
|  | My child is a good listener | .25 |  |  |  |
|  | My child takes things away when someone else is busy with them | .36 |  |  |  |
|  | My child seems to be on a different wavelength than others | .22 |  |  |  |
|  | My child talks about topics that only concern him/her | .35 |  |  |  |
| Emotional attunement | My child comforts others in times of sadness or pain | .47 | .79 | No obvious violations | Between ‘My child focuses his/her attention where others focus their attention’ and ‘My child comforts others in times of sadness or pain’ |
|  | My child offers help to others | .54 |  |  |  |
|  | My child plays appropriately with peers | .41 |  |  |  |
|  | My child focuses his/her attention where others focus their attention | .40 |  |  |  |
|  | My child is good at explaining to others what he/she means | .34 |  |  |  |
|  | My child lives in a world of their own | .44 |  |  |  |
| Emotion regulation | My child gets angry easily | .54 | .89 | Some deviation from monotonicity for items ‘My child is persistent’ and ‘My child finds it difficult to control himself’ | No local dependence |
|  | My child stays angry for a long time | .45 |  |  |  |
|  | My child is too sensitive | .30 |  |  |  |
|  | My child is sad | .40 |  |  |  |
|  | My child explodes seemingly out of nowhere | .56 |  |  |  |
|  | My child is in a good mood | .34 |  |  |  |
|  | My child is emotionally distant | .33 |  |  |  |
|  | My child can quickly change moods | .41 |  |  |  |
|  | My child must always get their way | .37 |  |  |  |
|  | My child is fussy about little things | .46 |  |  |  |
|  | My child is persistent | .35 |  |  |  |
|  | My child finds it difficult to control himself | .39 |  |  |  |
|  | My child gets frustrated when they cannot explain something | .47 |  |  |  |
|  | My child gets upset in situations with a lot of stimuli | .35 |  |  |  |
|  | My child cannot deal with conflict | .49 |  |  |  |
| Social cognition | My child does not understand the gist of a conversation | .46 | .79 | No obvious violations | Between ‘My child gives illogical reasons for what they do’ and ‘My child talks about things that are irrelevant’ |
|  | My child does not understand jokes | .34 |  |  |  |
|  | My child has difficulty following the flow of a conversation | .37 |  |  |  |
|  | My child understands how events are related (cause/effect) | .33 |  |  |  |
|  | My child talks about things that are irrelevant | .32 |  |  |  |
|  | My child has difficulty answering questions clearly | .41 |  |  |  |
|  | My child gives illogical reasons for what they do | .33 |  |  |  |
|  | My child can clearly express what they mean | .33 |  |  |  |
| Social motivation | My child does not look for comfort in sadness/pain | .27 | .82 | Some deviation from monotonicity for items ‘My child has difficulty making friends’ and ‘My child does not look for comfort in sadness/pain’ | Between ‘My child does not make contact with others on their own’ and, ‘My child prefers to be alone’; ‘My child does not participate when asked’; ‘My child is interested in other children/young people’ |
|  | My child prefers to be alone | .41 |  |  |  |
|  | My child does not make contact with others on their own | .44 |  |  |  |
|  | My child does not participate when asked | .30 |  |  |  |
|  | My child shares things with others | .27 |  |  |  |
|  | My child responds to requests | .31 |  |  |  |
|  | My child spontaneously tells something | .27 |  |  |  |
|  | My child is interested in other children/young people | .44 |  |  |  |
|  | My child collaborates with others | .41 |  |  |  |
|  | My child has difficulty making friends | .41 |  |  |  |
