# Supplementary figures and images for: A randomized controlled trial of the effects of dog-assisted versus robot dog-assisted therapy for children with autism or Down syndrome
Source: PLoS One. 2025 Mar 19;20(3):e0319939. doi: 10.1371/journal.pone.0319939 (PMC11922239; doi:10.1371/journal.pone.0319939)

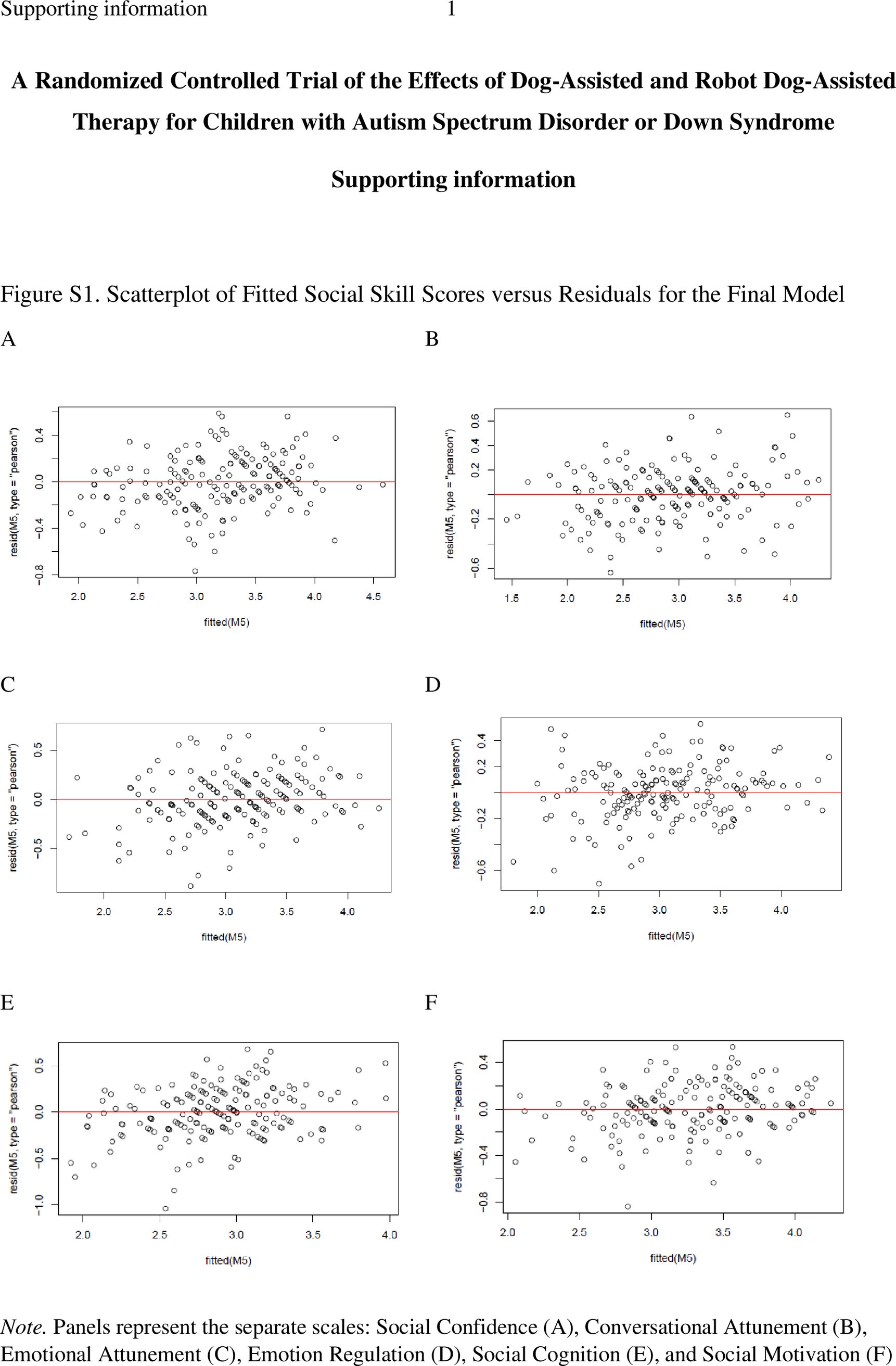

Supplement: S1 Fig — (TIF) [file pone.0319939.s004.tif]
