# Supplementary material for: A randomized controlled trial of the effects of dog-assisted versus robot dog-assisted therapy for children with autism or Down syndrome
Source: PLoS One. 2025 Mar 19;20(3):e0319939. doi: 10.1371/journal.pone.0319939 (PMC11922239; doi:10.1371/journal.pone.0319939)
Supplement: S2 Fig — (DOCX) [file pone.0319939.s005.docx]

**A Randomized Controlled Trial of the Effects of Dog-Assisted and Robot Dog-Assisted Therapy for Children with Autism Spectrum Disorder or Down Syndrome**

**Supporting information**

Figure S2. Q-Q plots of Within (left) and Between (right)-level residuals for the Final Model.

| 1. Social Confidence |  |
| --- | --- |
| 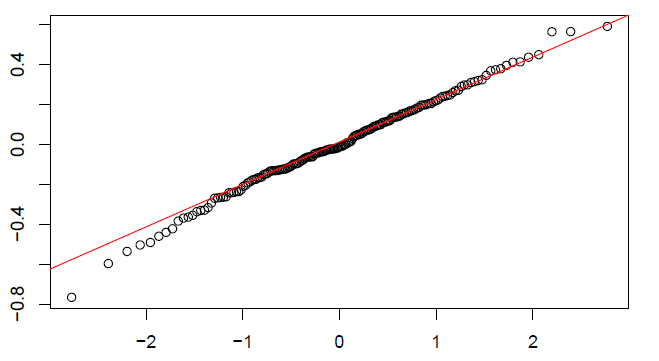 | 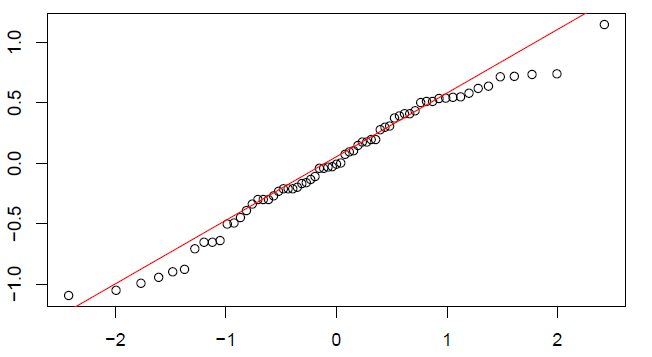 |
| 1. Conversational Attunement |  |
| 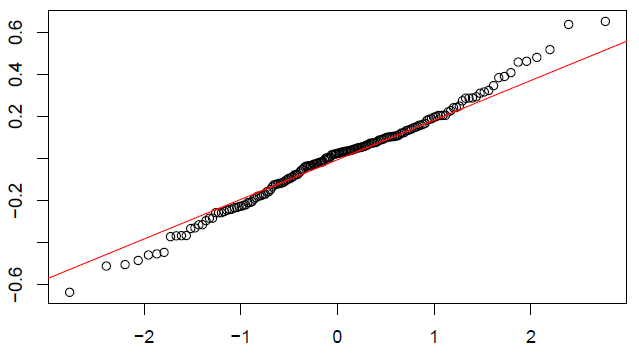 | 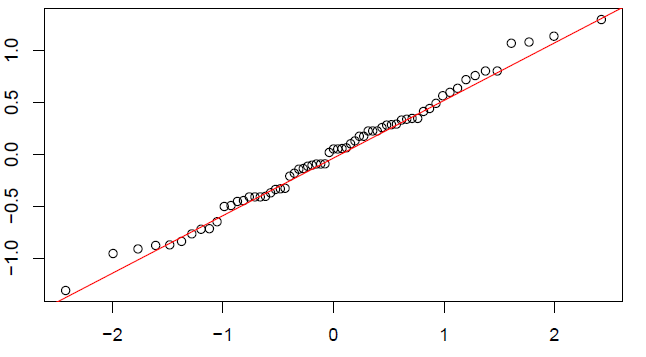 |
| 1. Emotional Attunement |  |
| 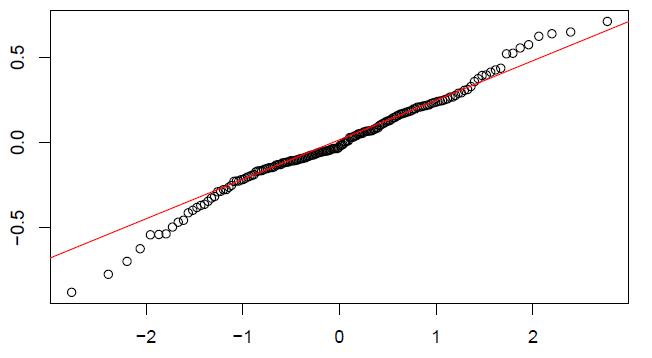 | 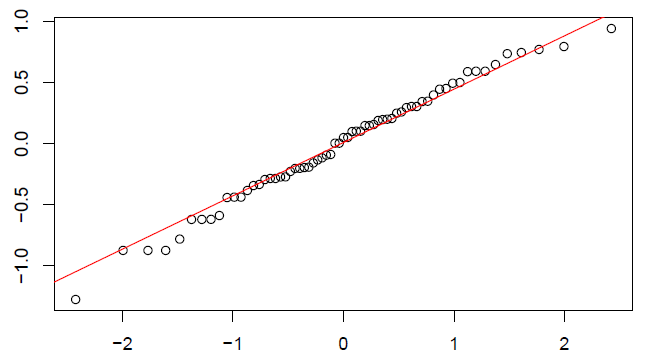 |
| 1. Emotion regulation |  |
| 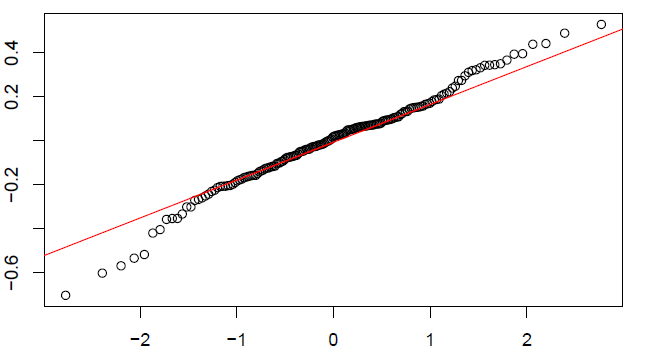 | 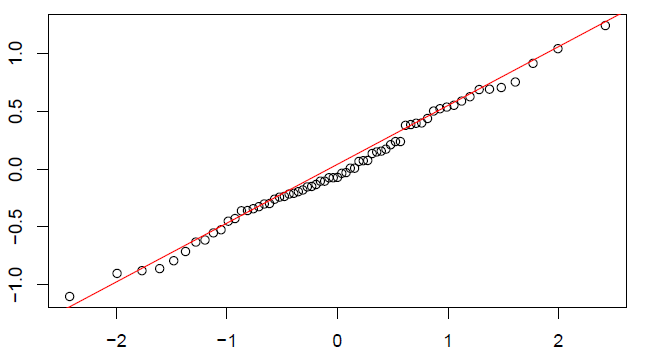 |
|  |  |
| 1. Social Cognition |  |
| 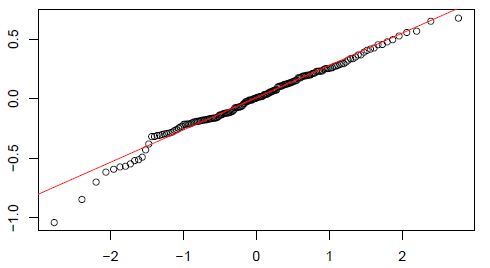 | 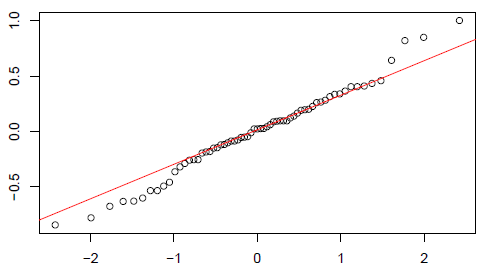 |
| 1. Social Motivation |  |
| 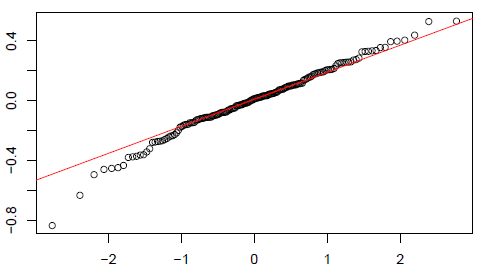 | 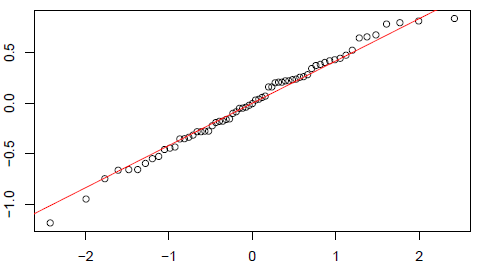 |
